# Application of six sigma management in surgical site infection reduction of patients with craniocerebral surgeries

**DOI:** 10.3389/fsurg.2026.1700976

**Published:** 2026-02-13

**Authors:** Jiayi Zhang, Xiaoxiao Li, Li Sun, Yanxia Gong

**Affiliations:** Department of Infection Management, Taihe County People's Hospital, Fuyang, Anhui, China

**Keywords:** neurosurgery, pathogenicbacteria, quality of life, six sigma management, surgical site infection

## Abstract

**Aim:**

Surgical site infection (SSI) is the most common hospital-acquired infection in neurosurgery. SSI after craniocerebral surgeries may cause serious harm to the prognosis of patients. Six Sigma management is a new type of management approach, and it reduces possible errors to the minimum by designing and monitoring processes, thereby achieving the highest level of quality and efficiency. However, the role of Six Sigma management in reducing SSI in patients with craniocerebral surgeries is not clear. Our study aimed to explore the effect of Six Sigma management in controlling surgical site infections in neurosurgery patients.

**Methods:**

This was a retrospective study. A total of 546 patients who underwent craniocerebral surgeries and were admitted to Taihe County People's Hospital from January 2021 to December 2021 were selected as the control group. A total of 550 patients who underwent craniocerebral surgeries and were admitted to Taihe County People's Hospital from January 2022 to December 2022 were selected as the study group. The control group adopted routine infection prevention and control methods. The study group adopted Six Sigma management methods. The incidence rate of SSIs, detection rate of pathogenic bacteria, hospital stay, nursing quality, quality of life and nursing satisfaction were compared in both groups.

**Results:**

Compared with the control group, the study group had lower incidence rate of SSIs, lower detection rate of pathogenic bacteria, shorter hospital stay, higher nursing quality scores, higher quality of life scores and better nursing satisfaction (*P* < 0.05 and *P* < 0.01).

**Conclusions:**

Six Sigma management can diminish the incidence rate of SSIs, diminish the detection rate of pathogenic bacteria, shorten the hospital stay, promote the nursing quality, promote the quality of life and enhance the nursing satisfaction of patients with craniocerebral surgeries.

## Introduction

Surgical site infection (SSI) refers to the infection that occurs at the incision site after the operation, including infections in the skin tissue, abdominal cavity, or pleural cavity (1). In neurosurgery, the incidence of postoperative SSI in patients undergoing craniocerebral surgery ranges from 1% to 8% (2). Craniocerebral surgery, as invasive treatment technique, will damage the protective barriers of the patient's hard and soft meninges during the treatment process (3). Moreover, the surface of the brain tissue has varying degrees of folds, while the intracranial tissues are rich in nutrients, providing an ideal environment for the growth and reproduction of pathogenic bacteria (4). If the operation is not performed properly, pathogenic bacteria will multiply in the brain, which may lead to infection (5). Postoperative SSI in neurosurgery patients can be devastating, increasing the risk of death by 2–11 times, and having an impact on the long-term disability of the patients after the surgery, prolonging their hospital stay by about one week (6). Therefore, medical staff should attach great importance to the prevention and control of surgical site infections in neurosurgery patients.

Hospital infection management is an important component of hospital medical quality and safety (7). The conventional infection prevention and control strategies have several shortcomings, including: (1) The lack of systematic data analysis makes it difficult to optimize the process and accurately evaluate the effectiveness; (2) The adopted uniform measures pay insufficient attention to individual differences; (3) The strategies are difficult to continuously improve, lack a feedback mechanism, and have a slow acceptance speed for new evidence, unable to meet the various needs of surgical site infection prevention and control for neurosurgical patients, thereby affecting the overall prevention and control effect (8). Therefore, while ensuring the proper implementation of basic infection control measures, how to conduct more in-depth and precise infection prevention for neurosurgery patients, and how to effectively implement SSI control for neurosurgery patients through management methods are the key points of hospital infection management (9).

Six Sigma management is a new type of management approach, and it reduces possible errors to the minimum by designing and monitoring processes, thereby achieving the highest level of quality and efficiency (10). Six Sigma management mainly encompasses three meanings: first, it is a quality standard and a pursuit goal; second, it is a set of scientific tools and management methods, using the improvement or design process to carry out the design and improvement of processes; third, it is an operation management strategy. Six Sigma management is a management process aimed at reducing costs, shortening cycles and improving patient satisfaction (11, 12). It is a management approach that can enhance the core competitiveness of hospitals and thereby strengthen the overall competitiveness of the hospitals (13). However, the role of Six Sigma management in controlling surgical site infections in neurosurgery patients remains unclear.

## Materials and methods

### Patients

This was a retrospective study. A total of 546 patients who underwent craniocerebral surgeries and were admitted to Taihe County People's Hospital from January 2021 to December 2021 were selected as the control group. A total of 550 patients who underwent craniocerebral surgeries and were admitted to Taihe County People's Hospital from January 2022 to December 2022 were selected as the study group. All patients signed the informed consent form, and this study was approved by the Ethics Committee of Taihe County People's Hospital. Inclusion criteria: (1) Patients who were hospitalized in the neurosurgery department and underwent craniotomy surgery; (2) Age more than 18 years old. Exclusion criteria: (1) Abnormal functions of the kidneys, heart and liver; (2) Other infectious diseases; (3) Impaired coagulation function or immune system; (4) Cognitive and communication impairments.

### Methods

The control group adopted routine infection prevention and control methods.

#### Before the operation

The patient was informed of the preoperative fasting and water deprivation time, which was usually 8–12 h of fasting and 4 h of water deprivation. Medical staff washed their hands according to the hospital's routine hygiene standards, put on surgical gowns and wore gloves. The operating room was cleaned and disinfected routinely, and the surfaces of the operating table and instrument table were wiped with regular disinfectants. The medical staff carried out their operations in accordance with the established ventilation and air purification procedures, without conducting any additional microbial monitoring or risk assessment.

#### During the operation

The surgical instruments were handled in accordance with the hospital's standard disinfection procedures. The transfer and use of the instruments followed the conventional aseptic operation principles. The surgeons performed craniocerebral surgeries in accordance with the conventional surgical operation norms. The movement of personnel in the operating room followed the hospital's regular management procedures.

#### After the operation

The patient's vital signs were monitored. The incision was treated with dressing changes according to a fixed schedule and method. Antimicrobial drugs were used appropriately based on the patient's specific condition.

The study group adopted Six Sigma management methods.

##### The definition stage

(a) Clarifying project objectives: The main project objective was to reduce the SSI for neurosurgery patients, and specific infection reduction targets were set. (b) Determining specific indicators for reducing infection rates: Identifying stakeholders such as gynecological surgeons, nurses, anesthesiologists, personnel from the infection control department, patients, and their families. Surgeons focused on the infection risks during surgical procedures, nurses were concerned about infection prevention before and after the surgery for patients, and patients and their family paid attention to surgical safety and post-operative recovery.

##### The measurement stage

(a) Collecting data: Basic information of the patients (age, weight, underlying diseases), type of surgery, duration of surgery, and preoperative hospital stay were collected. At the same time, the data of the surgical environment, such as the air cleanliness of the operating room, temperature and humidity, the cleanliness of surgical instruments, and the bacterial colony count on the hands of the surgical staff were monitored. In addition, the postoperative infection conditions, including the time of infection, type of infection, and types of microorganisms were recorded. (b) Determining key quality characteristics: The SSI was taken as a key quality characteristic, factors such as the quality of preoperative skin preparation, the disinfection effect of surgical instruments, and the implementation of aseptic operation by surgical staff were identified as the key determinants of the infection rate.

##### The analysis stage

An analysis of the high-risk factors for SSI was conducted, and the underlying causes such as incomplete sterilization of surgical instruments (due to equipment aging, non-standard procedures, insufficient time), and poor preoperative skin preparation of patients (lack of operation guidelines, insufficient training for medical staff) were identified.

##### The improvement stage

(a) Formulating improvement measures. Based on evidence-based medicine, a preoperative skin preparation plan was formulated. The skin was cleaned with a cleansing agent containing antibacterial components and was tested. The preoperative risk assessment was strengthened. The training of surgical staff was strengthened to ensure that their handwashing and other procedures comply with infection prevention and control requirements. The disinfection process for surgical instruments was optimized, considering the type and frequency of use to determine the disinfection method and duration. The aseptic operation was monitored in real time. The surgical environment was improved and the environmental parameters were adjusted according to the type of surgery and the patient's risk. An infection monitoring mechanism was established to monitor body temperature, white blood cell count, and symptoms at the surgical site. (b) Implementing improvement measures. Comprehensive improvement measures were implemented in the neurosurgery department. Medical staff were trained, operation procedures and norms were updated, and infection control equipment and supplies were provided. A project team was established to supervise the implementation.

##### The control stage

(a) Establishing a monitoring mechanism. The data related to patients' surgeries were continuously collected, the infection rates were regularly analyzed, the implementation of improvement measures were monitored, and the operation procedures and instrument disinfection processes of surgical staff were inspected. (b) Continuous improvement. Based on the results of the monitoring data, the existing issues were promptly adjusted and improved. If SSI failed to meet the expected reduction targets, the relevant data were re-analyzed to identify possible causes and the improvement measures were further optimized. The successful experiences and measures were standardized and incorporated into the routine neurosurgery management process of the hospital to ensure the continuous effectiveness of the infection control work at the surgical site.

### Observation indicators

The incidence rate of SSIs, detection rate of pathogenic bacteria, hospital stay, nursing quality, quality of life and nursing satisfaction were compared in both groups.

Secretions were collected from the surgical incision of the patient, and bacterial cultures were conducted on them. Pathogenic bacteria were then isolated and identified.

The nursing quality was evaluated using a self-developed nursing quality questionnaire, which covered 5 areas, with each area scoring between 5 and 15 points, and the total score ranging from 25 to 75 points. The score was positively correlated with the nursing quality.

The Short Form-36 (SF-36) scale was used to assess the quality of life of patients (14), which covered 8 dimensions: physical function, general health, role physical, energy, body pain, social function, mental health and emotional function. The score of each dimension ranged from 0 to 100. The higher the score, the better the quality of life.

The nursing satisfaction was assessed using a self-developed nursing satisfaction rating scale. The total score of the scale was 100 points. A score of 85 or above was considered very satisfied, a score between 60 and 84 was considered satisfied, and a score below 60 was considered dissatisfied. Total satisfaction = (number of satisfactory cases + number of very satisfactory cases)/total number of cases × 100%.

### Statistical analysis

GraphPad Prism 10.0 statistical software was employed for analyzing the data. The measurement data were exhibited by mean ± standard deviation (x ± s), and t-test was conducted for comparison. The counting data were exhibited as number and rate (%), and *χ*^2^ test was applied for comparison. *P* < 0.05 was considered as statistically significant.

## Results

As Table 1 displayed, no difference was seen in general data of patients between the two groups (*P* > 0.05), indicating comparability. To assess potential temporal confounding, we reviewed institutional records and confirmed that there were no major changes in neurosurgical team composition, antibiotic prophylaxis policy, or other hospital-wide infection control initiatives between the control period (2021) and the study period (2022).

**Table 1:** General data of patients in both groups.

Items	Control group (*n* = 546)	Study group (*n* = 550)	*χ*^2^/*t*	*P*
Gender			0.35	0.55
Male	353 (64.65)	365 (66.36)		
Female	193 (35.35)	185 (33.64)		
Age (years)	38.15 ± 5.43	38.23 ± 5.52	0.24	0.80
Cause of injury			0.17	0.98
Traffic accident	283 (51.83)	287 (52.18)		
High-altitude falling	104 (19.05)	106 (19.27)		
Violent beating	136 (24.91)	132 (24.00)		
Others	23 (4.21)	25 (4.55)		
Degree of injury			0.04	0.97
Mild	256 (46.89)	260 (47.27)		
Moderate	192 (35.16)	190 (34.55)		
Severe	98 (17.95)	100 (18.18)		

Compared with the control group, the study group had lower incidence rate of SSIs (*P* < 0.05, Table 2).

**Table 2:** Incidence rate of SSIs in both groups.

Groups	Cases	Surgical spot	Surface incision	Organ cavity	Total incidence rate
Control group	546	5 (0.92)	2 (0.37)	4 (0.73)	11 (2.02)
Study group	550	1 (0.18)	0 (0.00)	1 (0.18)	2 (0.36)
χ^2^					6.37
*P*					0.01

Compared with the control group, the study group had lower detection rate of pathogenic bacteria (*P* < 0.05, Table 3).

**Table 3:** Detection rate of pathogenic bacteria in both groups.

Groups	Cases	Acinetobacter baumannii	Klebsiella pneumoniae	Escherichia coli	Total detection rate
Control group	546	6 (1.10)	3 (0.55)	1 (0.18)	10 (1.83)
Study group	550	1 (0.18)	0 (0.00)	0 (0.00)	1 (0.18)
χ^2^					7.50
*P*					0.00

**Figure 1:**
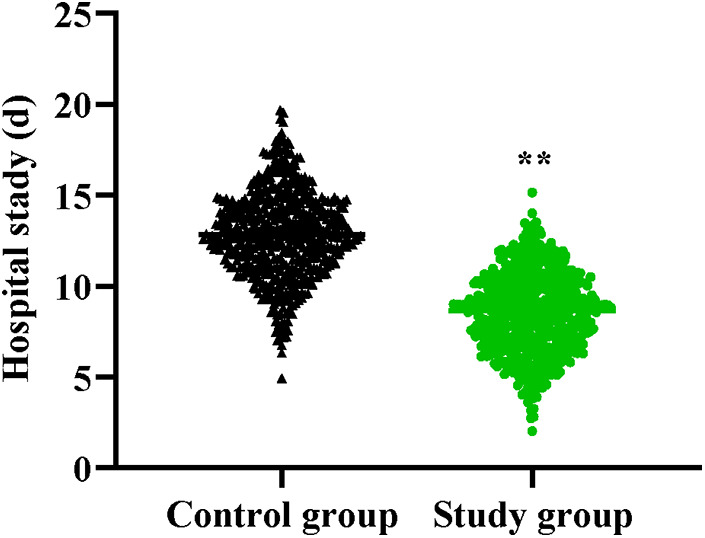
Hospital stay in both groups. **P < 0.01.

Compared to the control group, the study group had shorter hospital stay (*P* < 0.01, Figure 1).

**Figure 2:**
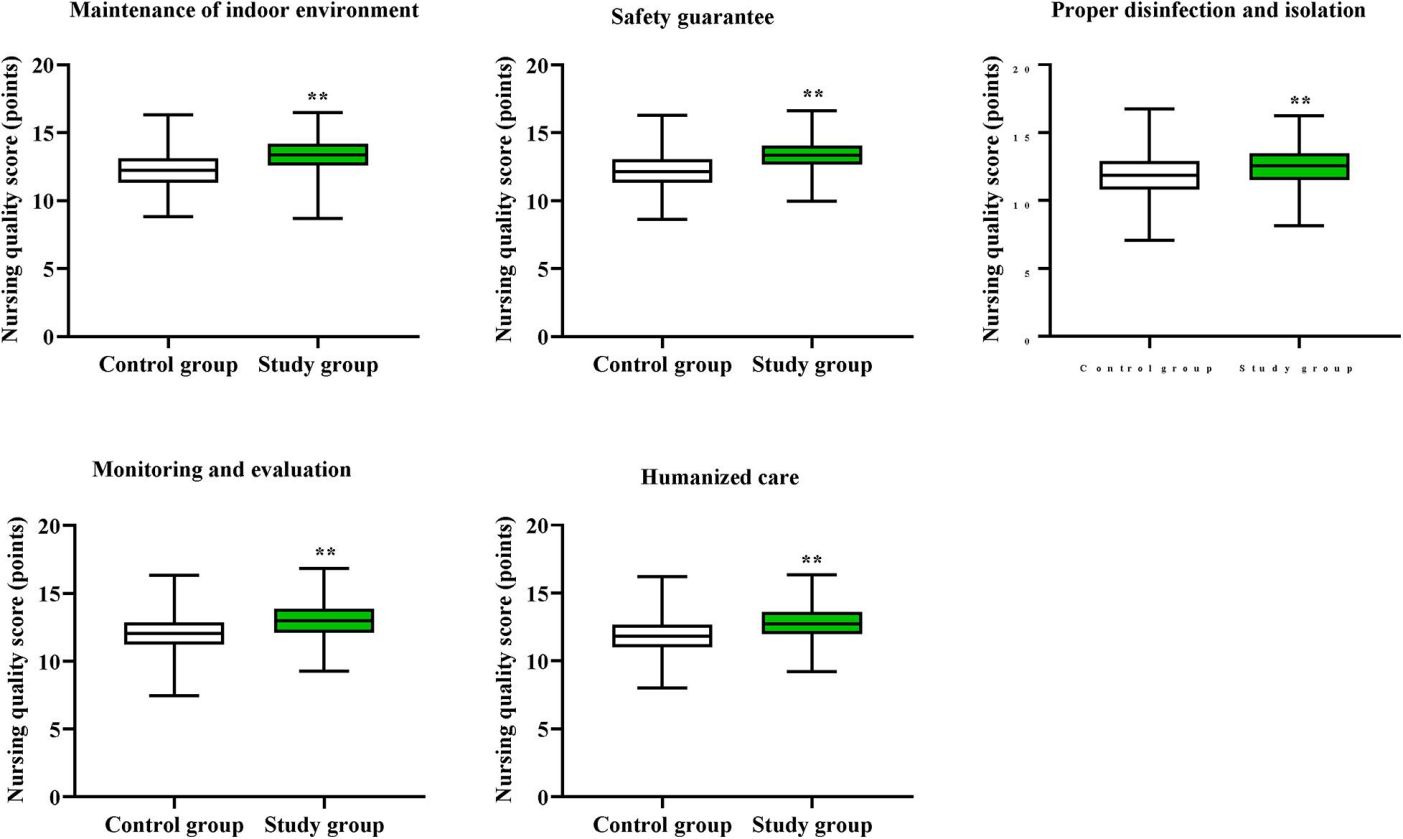
Nursing quality in both groups. **P < 0.01.

Compared to the control group, the study group had higher nursing quality scores in the aspects of maintenance of indoor environment, safety guarantee, proper disinfection and isolation, monitoring and evaluation, as well as humanized care (*P* < 0.01, Figure 2).

**Figure 3:**
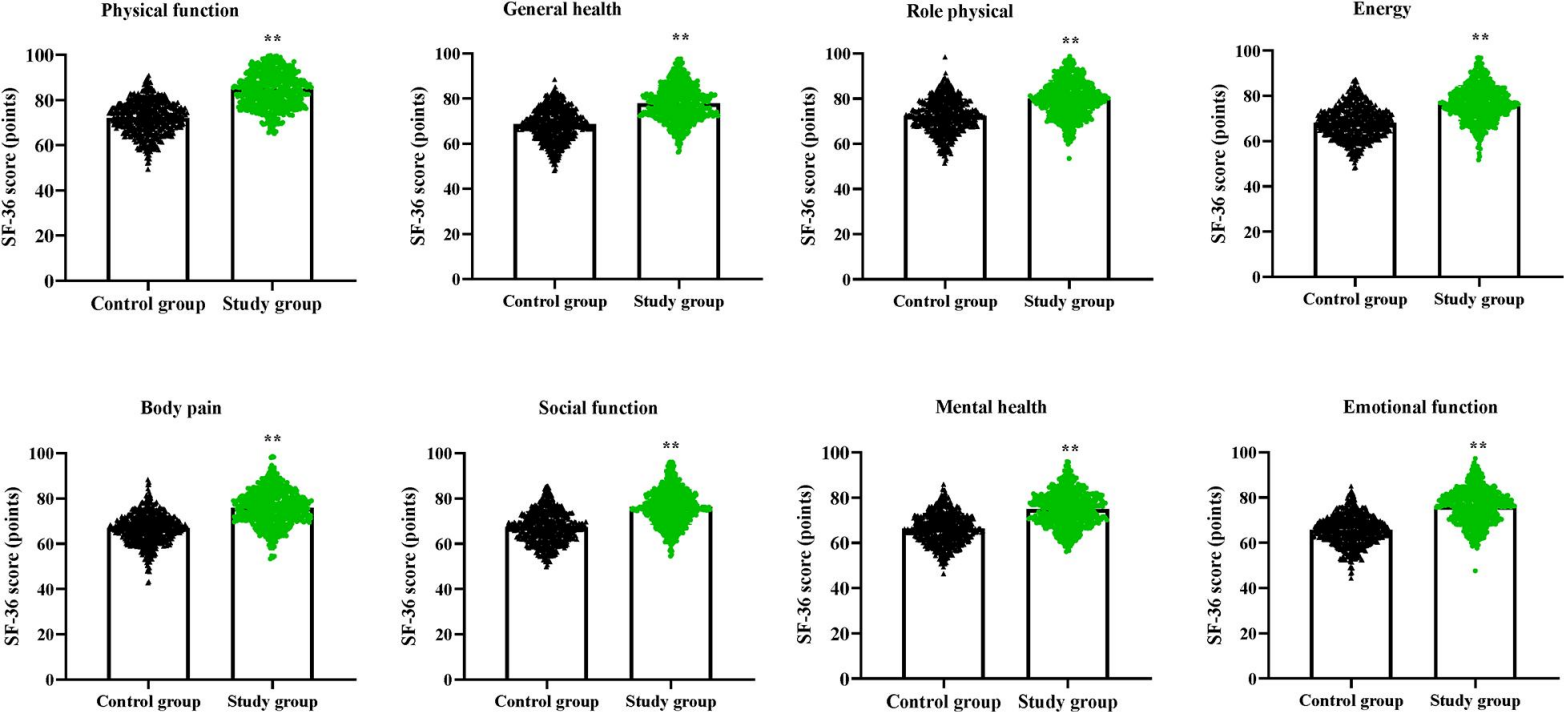
Quality of life in both groups. **P < 0.01.

Compared to the control group, the study group had higher SF-36 scores in the aspects of physical function, general health, role physical, energy, body pain, social function, mental health and emotional function (*P* < 0.01, Figure 3).

Compared to the control group, the study group had higher total nursing satisfaction (*P* < 0.05, Table 4).

**Table 4:** Nursing satisfaction in both groups.

Groups	Cases	Very satisfied	Satisfied	Dissatisfied	Total satisfaction rate
Control group	546	263 (48.17)	270 (49.45)	13 (2.38)	533 (97.62)
Study group	550	267 (48.55)	280 (50.91)	3 (0.54)	547 (99.45)
χ^2^					6.41
P					0.01

## Discussion

Craniocerebral surgeries are invasive procedures. During the treatment, the patient's epidermal tissue is damaged, causing the protective barriers such as the dura mater and arachnoid mater to lose their protective function (15). In addition, the surface of the brain tissue has folds, and the intracranial tissues have a reasonable proportion and abundant nutrition, which creates favorable conditions for the growth and reproduction of pathogenic bacteria (16). Once improper operations occur, it is very likely that bacteria will establish a colony in the brain tissue, leading to infection and causing a significant decline in the patient's physical functions and immune capacity (17). In patients undergoing craniocerebral surgeries, SSI is a common and serious complication, which not only affects the treatment outcome of the patients but also hinders their recovery (18). Postoperative SSIs in neurosurgical craniocerebral surgeries have the characteristics of rapid progression and acute onset, and factors such as the duration of postoperative drainage, the length of the surgery, and the patient's nutritional status can all be risk factors for infection (19). Moreover, due to the fact that patients are prone to invasion by highly resistant and highly viable pathogenic bacteria, the possibility of secondary infection is increased (20).

From a neurosurgical standpoint, cranioplasty is another cranial procedure associated with particularly high complication and infection rates. Large series of custom-made allograft and porous hydroxyapatite cranioplasties have reported overall complication rates in the range of approximately 16%–28%, with infection representing one of the most frequent and clinically relevant adverse events (21, 22). These data underscore cranioplasty as an unmet clinical need in terms of SSI prevention and suggest that structured management tools such as Six Sigma could be especially valuable for high-risk procedures. In the present study, elective cranioplasty procedures were not systematically captured and analyzed as a separate cohort, and our population mainly comprises adult patients undergoing craniotomy in the neurosurgery department. Nevertheless, our findings support the rationale for future studies that apply Six Sigma-based infection-control bundles specifically to cranioplasty and other high-risk neurosurgical interventions.

Six Sigma management originated in the late 1980s and is a new type of management approach developed by Motorola in the United States (23). It achieves the goals of enhancing enterprise quality, improving efficiency, reducing costs, and enhancing service levels by defining, measuring, analyzing, improving, and controlling (24). The Six Sigma management model has been applied in the transportation industry, electronics and electrical appliances, and healthcare to reduce the incidence of errors and improve the safety of treatment and care for patients (25). It has also been adopted in various fields such as manufacturing, energy, and chemical engineering (26). Nowadays, many hospitals in China have begun to experiment with the Six Sigma management model (27). The application of Six Sigma management in hospital management can effectively improve hospital management, reduce the occurrence of medical errors, enhance the level of medical services, and meet the modern people's demands for medical services (28). At the same time, Six Sigma is a specialized management methodology and its principles are not yet common knowledge among most clinicians, and formal belt certification can be costly. In our hospital, the Six Sigma initiative was coordinated by the infection management department and a small multidisciplinary project team, and front-line neurosurgical staff mainly participated through focused in-service training and updates of operating procedures embedded in routine continuing education rather than universal individual certification. This pragmatic, team-based approach may help other institutions adopt key Six Sigma tools without incurring prohibitive training costs for all staff.

The results of our study indicated that compared with the control group, the study group had lower incidence rate of SSIs and lower detection rate of pathogenic bacteria. The reason is that Six Sigma identifies problems and opportunities for improvement by collecting and analyzing data, and places great emphasis on the continuous optimization of processes: (1) By analyzing historical data and the current process, the key factors causing SSI can be determined, and targeted preventive measures can be formulated accordingly. (2) By optimizing aspects such as the environment of the operating room, surgical procedures, patient preparation, and post-operative care, the risk of SSIs and pathogenic bacteria can be reduced. (3) By establishing and adhering to standardized operation procedures, standardized preoperative preparations, aseptic operations, and postoperative management, the rate of SSIs can be significantly reduced, and the proliferation of pathogenic bacteria can also be prevented. (4) By providing regular training to medical staff, ensuring that they are familiar with the latest infection control knowledge and skills, the risk of SSIs and the proliferation of pathogenic bacteria caused by improper operations can be reduced. (5) By educating patients about the importance of preoperative preparation and postoperative care, patients' compliance can be improved, further lowering the risk of SSIs and the proliferation of pathogenic bacteria. (6) By regularly inspecting and evaluating the effectiveness of infection control measures, problems can be identified and adjusted in a timely manner, which can effectively reduce the incidence of SSI the proliferation of pathogenic bacteria in neurosurgery patients. Consistently, Feng et al. suggested that the Six Sigma method significantly decreased catheter-related bloodstream infections incidence from 12.79 to 2.32 per 1,000 catheter-days among non-ICU hemodialysis patients (29).

The results of our study indicated that compared to the control group, the study group had shorter hospital stay. The reason is that based on infection control strategy of Six Sigma management, the incidence of SSIs is low. Postoperative SSI is an important factor in prolonging hospital stay, and reducing the occurrence of SSIs can enable patients to recover their bodies more quickly, thereby shortening the hospital stay. In line with our finding, Improta et al. indicated that the application of Six Sigma method significantly decreased the length of stay of stroke patients (30). In addition, our study manifested that, compared to the control group, the study group had higher nursing quality scores, SF-36 scores and higher total nursing satisfaction, suggesting that Six Sigma management could promote the nursing quality, promote the quality of life and enhance the nursing satisfaction of patients with craniocerebral surgeries. Similarly, Bertolaccini et al. proposed that the Six Sigma approach increased patient satisfaction of patients who underwent pulmonary intervention (31).

The results of this study are applicable to the patient population undergoing neurosurgery. The hospital needs to have certain basic conditions, such as a complete infection monitoring system, a medical team capable of implementing standardized operating procedures, and managers who have a certain understanding and application ability of Six Sigma management and evidence-based medicine. Meanwhile, this strategy is applicable to a wide range of neurosurgical procedures. However, for some patients with particularly complex neurosurgical diseases, further adjustments and optimizations may be necessary based on the individual characteristics of the patients. While our findings suggest that Six Sigma management may be beneficial in neurosurgical SSI prevention, they should be interpreted with caution given that this was a single-center retrospective study conducted in the neurosurgery department of a county-level hospital in China and focused mainly on adult trauma patients undergoing craniotomy. As such, the external validity of our results to other neurosurgical populations (e.g., tumor or vascular surgery), to other healthcare systems and to different resource settings may be limited. Our data add to the growing body of evidence supporting Six Sigma management in infection prevention, but multicenter prospective studies in diverse neurosurgical settings are needed before definitive conclusions can be drawn.

However, this study also has some limitations. First, the time span was relatively short (two consecutive years), which may not fully reflect the long-term and sustained effects of Six Sigma management. Second, although the overall sample size exceeded 1,000 patients, the absolute number of SSI events was small, which may limit statistical power and increase the uncertainty of effect estimates, and it also prevented more detailed subgroup analyses or reliable multivariable analyses adjusting for known SSI risk factors (e.g., operation time, injury severity). Future studies with larger cohorts should incorporate such adjusted analyses to confirm the independent effect of Six Sigma management. Third, the retrospective, single-center design makes it difficult to exclude unmeasured confounding factors, such as changes in case mix, staff composition or concurrent infection-control initiatives, that might have contributed to the observed improvements. Fourth, we did not comprehensively assess potential influencing factors such as patients' lifestyles, genetic factors, and the surrounding environment. Therefore, in the subsequent clinical work, we will conduct long-term follow-up and comprehensive clinical assessment of the patients to ensure the credibility of the results.

## Conclusions

Six Sigma management can diminish the incidence rate of SSIs, diminish the detection rate of pathogenic bacteria, shorten the hospital stay, promote the nursing quality, promote the quality of life and enhance the nursing satisfaction of patients with craniocerebral surgeries.

## Data Availability

The original contributions presented in the study are included in the article/Supplementary Material, further inquiries can be directed to the corresponding author.

## References

[1] Ierano C Hall L James R. Surgical site infection prophylaxis: what have we learned and are we making progress? Curr Opin Infect Dis. (2023) 36:450–61. 10.1097/QCO.0000000000000970 37755394

[2] Yasuhara T Date I. [Surgical site infection(SSI)in neurosurgery]. No Shinkei Geka. (2021) 49:1093–104. 34615769 10.11477/mf.1436204493

[3] Yue Z Zhi X Bi L Zhao L Ji J. Treatment and prognostic risk factors for intracranial infection after craniocerebral surgery. Neurosurg Rev. (2023) 46:199. 10.1007/s10143-023-02106-0 37568062

[4] Xiong X Xie Y Li B Yin C Hu K. Treatment of intracranial infection by extensively drug-resistant Acinetobacter Baumannii after craniocerebral surgery. J Craniofac Surg. (2024) 35:e673–5. 10.1097/SCS.0000000000010542 39418529

[5] Wang YJ Zhao ZH Lu SK Wang GL Ma SJ Wang LH Analysis of risk factors, pathogenic bacteria characteristics, and drug resistance of postoperative surgical site infection in adults with limb fractures. Chin J Traumatol. (2024) 28(4):241–51. 10.1016/j.cjtee.2024.04.007 38811319 PMC12281841

[6] Duquette E Bhatti P Sur S Felbaum DR Dowlati E. History and use of antibiotic irrigation for preventing surgical site infection in neurosurgery: a scoping review. World Neurosurg. (2022) 160:76–83. 10.1016/j.wneu.2022.01.098 35101611

[7] Liu W Guo T Li H Zhao Y Zhang K Hai Y Healthcare-associated infection prevention and control management in a tertiary hospital and an overall evaluation. Ann Palliat Med. (2020) 9:1536–44. 10.21037/apm-20-65 32692198

[8] Chen Q Zu ZY Jiang MD Lu L Lu GM Zhang LJ. Infection control and management strategy for COVID-19 in the radiology department: focusing on experiences from China. Korean J Radiol. (2020) 21:851–8. 10.3348/kjr.2020.0342 32524785 PMC7289699

[9] Han DS Tan M Xie SL Bo Y Tu YR. Application of plan-do-check-action cycle in the management of reducing surgical site infection rate of craniotomy in neurosurgery. Neurosurg Rev. (2025) 48:361. 10.1007/s10143-025-03526-w 40216621

[10] Thakur V Akerele OA Randell E. Lean and six sigma as continuous quality improvement frameworks in the clinical diagnostic laboratory. Crit Rev Clin Lab Sci. (2023) 60:63–81. 10.1080/10408363.2022.2106544 35978530

[11] Al Nemari M Waterson J. The Introduction of robotics to an outpatient dispensing and medication management process in Saudi Arabia: retrospective review of a pharmacy-led multidisciplinary six sigma performance improvement project. JMIR Hum Factors. (2022) 9:e37905. 10.2196/37905 36222805 PMC9597422

[12] Carsten BF Bhandari P Fortney BJ Wilmes DS Nelson CM Brien AL Quality improvement initiative to improve communication domains of patient satisfaction in a regional community hospital with six sigma methodology. BMJ Open Qual. (2023) 12:e002306. 10.1136/bmjoq-2023-002306 38160018 PMC10759047

[13] Van Dalen A Strandbygaard J Van Herzeele I Boet S Grantcharov TP Schijven MP. Six sigma in surgery: how to create a safer culture in the operating theatre using innovative technology. Br J Anaesth. (2021) 127:817–20. 10.1016/j.bja.2021.08.023 34593216

[14] Essex R Govintharjah P Issa R Kalocsányiová E Lakika D Markowski M Health related quality of life amongst refugees: a meta analysis of studies using the SF-36. J Immigr Minor Health. (2024) 26:925–35. 10.1007/s10903-024-01615-4 38958897 PMC11413143

[15] Fu Y Jin Z. Effects of dexmedetomidine on cognitive function, oxidative stress and brain protection in patients undergoing craniocerebral surgery. Actas Esp Psiquiatr. (2024) 52:19–27. 10.62641/aep.v52i1.1551 38454897 PMC10926013

[16] Li H Liu K Li H Gu J Yao L. Effect of intravenous anesthesia with remimazolam besylate on hemodynamics and neuroprotection in patients undergoing surgery for craniocerebral injury. Ann Ital Chir. (2025a) 96:543–9. 10.62713/aic.3867 40234226

[17] Liang S Fan X Chen F Liu Y Qiu B Zhang K Chinese guideline on the application of anti-seizure medications in the perioperative period of supratentorial craniocerebral surgery. Ther Adv Neurol Disord. (2022) 15:17562864221114357. 10.1177/17562864221114357 35992894 PMC9386849

[18] Li Y Gao L Fan S. The characteristics of surgical site infection with class I incision in neurosurgery. BMC Surg. (2025b) 25:97. 10.1186/s12893-025-02825-9 40075338 PMC11900087

[19] Berghmans M De Ghellinck L De Greef J Di Santo M Ribeiro Vaz JG Zech F Outcome of patients with surgical site infection after craniotomy. Surg Infect (Larchmt). (2022) 23:388–93. 10.1089/sur.2021.260 35333641

[20] Hasegawa H Saito N. [Surgical site infection following craniotomies]. No Shinkei Geka. (2022) 50:1008–16. 36128816 10.11477/mf.1436204660

[21] Fricia M Nicolosi F Ganau M Cebula H Todeschi J Santin MDN Cranioplasty with porous hydroxyapatite custom-made bone flap: results from a multicenter study enrolling 149 patients over 15 years. World Neurosurg. (2019) 121:160–5. 10.1016/j.wneu.2018.09.199 30315976

[22] Ganau M Cebula H Fricia M Zaed I Todeschi J Scibilia A Surgical preference regarding different materials for custom-made allograft cranioplasty in patients with calvarial defects: results from an internal audit covering the last 20 years. J Clin Neurosci. (2020) 74:98–103. 10.1016/j.jocn.2020.01.087 32033859

[23] Coughlin K Posencheg MA. Common quality improvement methodologies including the model for improvement, lean, and six sigma. Clin Perinatol. (2023) 50:285–306. 10.1016/j.clp.2023.02.002 37201982

[24] Kannan N Ramalingam K Ramani P. Revolutionising quality management in the oral pathology laboratory: a deep dive into the six sigma methodology. Cureus. (2024) 16:e52651. 10.7759/cureus.52651 38380190 PMC10877558

[25] Pope J Boyle J Worrall M. Airway management in paediatric emergencies outside of an intensive care setting: a quality improvement project using lean/six sigma methodology. Arch Dis Child Educ Pract Ed. (2023) 108:463–6. 10.1136/archdischild-2023-325329 37164482

[26] Hernández-Lara AB Sánchez-Rebull MV Niñerola A. Six sigma in health literature, what matters? Int J Environ Res Public Health. (2021) 18:8795. 10.3390/ijerph18168795 34444542 PMC8394710

[27] Zimmermann GDS Siqueira LD Bohomol E. Lean six sigma methodology application in health care settings: an integrative review. Rev Bras Enferm. (2020) 73:e20190861. 10.1590/0034-7167-2019-0861 33338158

[28] Jiang P Liu Y Gu HY Li QX Xue LB. Implementation of six sigma management to standardize surgical hand disinfection practices. BMC Surg. (2025) 25:118. 10.1186/s12893-025-02854-4 40148851 PMC11948759

[29] Feng X Huang Q Yuan L Lu F Deng R Xia P. Reducing catheter-related bloodstream infections using lean six sigma methodology. BMC Health Serv Res. (2024) 24:1121. 10.1186/s12913-024-11527-6 39334368 PMC11430130

[30] Improta G Borrelli A Triassi M. Machine learning and lean six sigma to assess how COVID-19 has changed the patient management of the Complex operative unit of neurology and stroke unit: a single center study. Int J Environ Res Public Health. (2022) 19:5215. 10.3390/ijerph19095215 35564627 PMC9103695

[31] Bertolaccini L Rizzardi G Filice MJ Terzi A. ’Six sigma approach'—an objective strategy in digital assessment of postoperative air leaks: a prospective randomised study. Eur J Cardiothorac Surg. (2011) 39:e128–132. 10.1016/j.ejcts.2010.12.027 21316980

